# Neuropathological evaluation of the Boston Criteria v2.0 for cerebral amyloid angiopathy in Alzheimer's disease dementia

**DOI:** 10.1002/alz70856_105538

**Published:** 2026-01-07

**Authors:** Mario Ricciardi, Iván Burgueño‐García, Elizabeth Valeriano‐Lorenzo, María Ascensión Zea‐Sevilla, Meritxell Valentí, Belén Frades, Isabel López Torres, Marta Anton‐Moreno, Francisco J. López‐González, Paloma Ruiz‐Valderrey, Laura Saiz, Alicia Uceda‐Heras, Linda Zhang, Mabel Torres Llacsa, Eva Alfayate, Marta Molero Cartón, María José López‐Martínez, Teodoro del Ser, Michel J. Grothe, Alberto Rabano, Pascual Sánchez‐Juan

**Affiliations:** ^1^ Alzheimer's Center Reina Sofia‐CIEN Foundation, Madrid, Madrid, Spain; ^2^ CIEN Foundation, Reina Sofia Alzheimer Center, ISCIII, Madrid, Madrid, Spain

## Abstract

**Background:**

Cerebral amyloid angiopathy (CAA) is closely related and coexists with Alzheimer's disease (AD) and is considered a major risk factor for the development of ARIA in patients receiving anti‐amyloid therapies. Brain magnetic resonance imaging (MRI) is the standard method for assessing the presence of CAA, but its performance has been poorly studied in this population. Our objective was to evaluate the diagnostic performance of the Boston criteria version 2.0 for the diagnosis of CAA in a cohort of individuals diagnosed with advanced AD with neuropathological confirmation.

**Method:**

We included 63 individuals from the VARS cohort of the Alzheimer´s Centre Reina Sofía ‐ CIEN Foundation, a clinicopathological cohort of dementia patients, with no history of intracranial haemorrhage. All had undergone at least one antemortem MRI with T1, T2, T2 Flair and GRE sequences, and a brain autopsy with evaluation of CAA using the modified Vonsattel scale. The performance of the Boston v2.0 criteria was evaluated by comparison with neuropathologically verified CAA.

**Result:**

The mean age at MRI was 84.7 ± 7 years and the median time from MRI to death was 2.5 years [0.6‐4.5]. 54 were women (86%). 46 (73%) individuals had neuropathologically confirmed CAA (i.e. moderate or severe CAA). 38 (60,3%) subjects did not meet the Boston v2.0 criteria and 45% of them had moderate or severe CAA. The sensitivity and specificity of the Boston v2.0 criteria were 40.8% [95% CI 27%‐55.8%] and 64.2% [95% CI 35.1%‐87.2%] respectively for the diagnosis of probable CAA (AUC 0.52), and 83.7% [95% CI 70.3%‐92.7%] and 35.7% [95% CI 12.8%‐64.9%] for the diagnosis of possible CAA (AUC 0.51).

**Conclusion:**

The Boston v2.0 criteria have low accuracy in patients with AD dementia. New biomarkers are needed to improve the diagnosis of CAA in this population in order to improve the safety of treatment with anti‐amyloid drugs.

